# Inhibition of Aldose Reductase by *Gentiana lutea* Extracts

**DOI:** 10.1155/2012/147965

**Published:** 2012-07-15

**Authors:** Chandrasekhar Akileshwari, Puppala Muthenna, Branislav Nastasijević, Gordana Joksić, J. Mark Petrash, Geereddy Bhanuprakash Reddy

**Affiliations:** ^1^Biochemistry Division, National Institute of Nutrition, Tarnaka, Jamai-Osmania, Hyderabad 500 007, India; ^2^Vinca Institute of Nuclear Sciences, University of Belgrade, 11001 Belgrade, Serbia; ^3^Department of Ophthalmology, University of Colorado School of Medicine, Aurora, CO 80045, USA

## Abstract

Accumulation of intracellular sorbitol due to increased aldose reductase (ALR2) activity has been implicated in the development of various secondary complications of diabetes. Thus, ALR2 inhibition could be an effective strategy in the prevention or delay of certain diabetic complications. *Gentiana lutea* grows naturally in the central and southern areas of Europe. Its roots are commonly consumed as a beverage in some European countries and are also known to have medicinal properties. The water, ethanol, methanol, and ether extracts of the roots of *G. lutea* were subjected to *in vitro* bioassay to evaluate their inhibitory activity on the ALR2. While the ether and methanol extracts showed greater inhibitory activities against both rat lens and human ALR2, the water and ethanol extracts showed moderate inhibitory activities. Moreover, the ether and methanol extracts of *G. lutea* roots significantly and dose-dependently inhibited sorbitol accumulation in human erythrocytes under high glucose conditions. Molecular docking studies with the constituents commonly present in the roots of *G. lutea* indicate that a secoiridoid glycoside, amarogentin, may be a potential inhibitor of ALR2. This is the first paper that shows *G. lutea* extracts exhibit inhibitory activity towards ALR2 and these results suggest that *Gentiana* or its constituents might be useful to prevent or treat diabetic complications.

## 1. Introduction

According to the latest WHO estimates, currently approximately 200 million people all over the world are suffering from diabetes. This may increase to at least 350 million by the year 2025, which could have a severe impact on human health [1]. Prolonged exposure to chronic hyperglycemia in diabetes can lead to various complications affecting the cardiovascular, renal, neurological, and visual systems [2]. Although mechanisms leading to diabetic complications are not completely understood, many biochemical pathways associated with hyperglycemia have been implicated [2]. Among these, the polyol pathway has been extensively studied [3].

Aldose reductase (ALR2; EC: 1.1.1.21) belongs to aldo-keto reductases (AKR) super family. It is the first and rate-limiting enzyme in the polyol pathway where it reduces glucose to sorbitol utilizing NADPH as a cofactor. Subsequently, sorbitol dehydrogenase catalyzes the conversion of sorbitol to fructose, thus constituting the polyol pathway [3]. Accumulation of sorbitol leads to osmotic swelling, changes in membrane permeability, and also oxidative stress culminating in tissue injury [4]. Experimental animal models suggest that the inhibition of ALR2 could be effective in prevention of certain complications [5]. A number of ALR2 inhibitors (ARI) have been developed for diabetic complications; however, none of them has achieved worldwide use because of limited efficacy or undesirable side effects [6–9]. Largely, two chemical classes of ARI have been tested in phase III trials. While carboxylic acid inhibitors (zopolrestat, ponalrestat, and tolrestat) have shown poor tissue permeability and are not very potent *in vivo*, spiroimide (spirohydantoin) inhibitors such as sorbinil penetrate tissues more efficiently but many have been associated with skin reactions and liver toxicity [4, 7, 8, 10]. Thus, there is a need for developing and evaluating new ARI considering efficacy, selectivity, and safety issues. We have previously reported, using *in vitro, ex vivo*, and in animal models, that some common dietary sources such as spices, fruits and vegetables have the ARI potential [11–15].

*Gentiana lutea*, commonly known as bitter wort, is a plant belonging to the family *Gentianaceae*, which grows in the grassy alpine and subalpine pastures, usually on calcareous soils native to the mountains of central and southern Europe [16]. The roots of* G. lutea *are commonly consumed as a beverage in some of the European countries and are also known to have medicinal properties due to the presence of bitter glycosides. However, so far ARI potential of any of the extracts of *Gentiana* has not been reported. Therefore, in the present study we have assessed the ARI potential of various extracts of *G. lutea* and report that secoiridoid glycosides and xanthone principles of *G. lutea *may be the potent inhibitors of ALR2. Further, these results are supported by molecular docking studies. We also investigated the effects of these extracts on intracellular sorbitol accumulation in red blood cells (RBC) under *ex vivo* high glucose conditions which reinforce the ARI potential of *Gentiana*.

## 2. Materials and Methods

### 2.1. Materials

Ammonium sulphate, D-glucose, DL-glyceraldehyde, dimethylsulfoxide, EDTA, glycine, lithium sulphate, 2-mercaptoethanol, methyl orange, NADPH, NADP, perchloric acid, sorbitol, sorbitol dehydrogenase, sucrose, and Tris-HCl were obtained from Sigma-Aldrich (St. Louis, MO). All other chemicals and solvents were of analytical grade and were obtained from local companies.

### 2.2. Preparation of *G. lutea* Extracts

*G. lutea *roots used for ethanol, methanol, ether, and water extracts preparation were purchased from “Dr. Josif Pancic”, Institute of Medicinal Plant Research Belgrade, Serbia. Five grams of dried *G. lutea* roots were powdered and extracted with 100 mL of methanol, ethanol or ether, at room temperature, for 48 h, with occasional shaking. After filtering, the extracts were dried using a rotary evaporator (Buchi R-210/215) at 30–35°C, with yield of 37.8% of crude extract for methanol, 4.63% for ether, 31.76% for ethanol and 18.33% for water. The stock solutions containing 50 mg/mL methanol, 50 mg/mL ethanol, 100 mg/mL water and 40 mg/mL ether extracts were prepared immediately before use.

### 2.3. Separation and Characterization of Extracts from *G. lutea*

Chromatographic separations of the extracts were performed using a BEH C18 column (1.7 *μ*m, 100 × 2.1 mm) on a Waters Acquity UPLC system, equipped with UV visible detector. Running of extracts was performed in gradient with mobile phase consisting of solvent (a) trifluoroacetic acid (0.1% v/v in water) and (b) acetonitrile/methanol mixture (85 : 15, v/v). The eluent flow rate was 0.3 mL min^−1^, the injection volume was 10 *μ*L and detection was at 250 nm. MALDI-TOF mass spectra of UPLC fractions were acquired on a Voyager Biospectrometry DEPro Workstation (Perseptive Biosystems, Framingham, MA, USA). The system utilizes a 20 Hz pulsed nitrogen laser emitting at 337 nm.

### 2.4. Preparation of Rat Lens ALR2

Crude ALR2 was prepared from rat lens as described previously [11]. Lenses were homogenized in 9 volumes of 100 mM potassium phosphate buffer, pH 6.2. The homogenate was centrifuged at 15,000 ×g for 30 min at 4°C and the resulting supernatant was used as the source of ALR2.

### 2.5. Expression and Purification of Recombinant Human ALR2

Recombinant human ALR2 was overexpressed in *Escherichia coli* and purified from bacterial cultures essentially as described previously [14, 17] with a minor modification. Chromatography over AffiGel Blue (Bio-Rad) affinity matrix was used in final purification step.

### 2.6. Aldose Reductase (ALR2) Assay

ALR2 activity was assayed as described previously [11]. The assay mixture in 1 mL contained 50 mM potassium phosphate buffer, pH 6.2, 0.4 M lithium sulphate, 5 mM 2-mercaptoethanol, 10 mM DL-glyceraldehyde, 0.1 mM NADPH, and enzyme preparation (rat lens or recombinant enzyme). Appropriate blanks were employed for corrections. The assay mixture was incubated at 37°C and the reaction was initiated by the addition of NADPH at 37°C. The change in the absorbance at 340 nm due to NADPH oxidation was followed in a spectrophotometer (Lamda-35, Perkin-Elmer, Shelton, USA).

### 2.7. Inhibition Studies

For inhibition studies concentrated stocks of *G. lutea* extracts were prepared in water/DMSO. Various concentrations of these extracts were added to the ALR2 assay mixture and incubated for 5 min before initiating the reaction by NADPH as described above. The percent inhibition with test compounds was calculated considering the ALR2 activity in the absence of inhibitor as 100%. The concentration of each test sample giving 50% inhibition (IC_50_) was determined by nonlinear regression analysis of log concentration of extract versus percentage inhibition.

### 2.8. Molecular Docking

Molecular docking was done by discovery (Discover 2.7) package from (Biosystems Technologies, San Diego, CA, USA), on an O2 (R12000) Workstation (Silicon Graphics, Mountain View, CA, USA) and GOLD 3.1 (Genetic Optimized for Ligand Docking). All ligands were minimized and least energy conformations were taken for docking studies. Crystal structure of ALR2 was downloaded from Brookhaven data bank (PDB: 1PWM) and protein structure minimized by using charmM force field. All water molecules were removed. Docking was done by discovery ligandfit module in a protein-created sphere of about 12 Å around the active site. After docking, poses were viewed by DS Viewer and PoseView (Biosolve IT) and LigandScout.

### 2.9. *In Vitro* Incubation of RBC

Five mL blood was collected from healthy male volunteers on overnight fasting in heparinized tubes. The study protocols were approved by Institutional Ethics Committee. Red blood cells were separated by centrifugation and washed thrice with isotonic saline. Washed RBC were suspended in Kreb's-ringer bicarbonate buffer, pH 7.4 (preequilibrated with 5% CO_2_) and incubated at 37°C in presence of 5% CO_2_ for 3 h under normal (5.5 mM) and high glucose (55 mM) conditions [13, 14]. The effect of *G. lutea *extracts on sorbitol accumulation was evaluated by incubating RBC with different concentrations of extracts.

### 2.10. Estimation of Sorbitol in RBC

At the end of the incubation period, RBC was homogenized in 9 volumes of 0.8 M perchloric acid. The homogenate was centrifuged at 5,000 ×g at 4°C for 10 min and the pH of the supernatant was adjusted to 3.5 with 0.5 M potassium carbonate. The sorbitol content of the supernatant was measured by fluorometric method as described previously [18] using a spectrofluorometer (Jasco-FP 6500, Japan).

## 3. Results

### 3.1. Characterization of the Extracts from *G. lutea*

**Figure 1:**
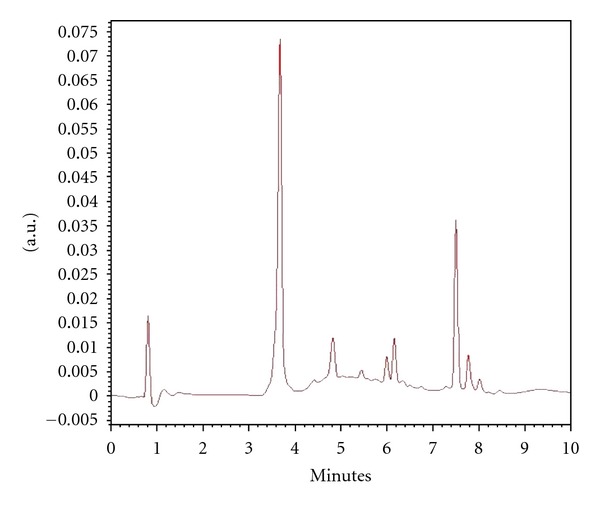
UPLC chromatogram of 0.1 mg/mL methanol extract of *Gentiana lutea*.

The UPLC chromatogram indicated the presence of at least 10 compounds in methanol extract (Figure 1). Partial characterization of the methanol extract was performed using MALDI-TOF, after its separation and fractionation by UPLC. MALDI-TOF analysis of methanol extract indicated the possible presence of the following compounds: gentisin, bellidifolin-8-O-glucoside, demethylbellidifolin-8-O-glucoside, isovitexin, swertiamarin, amarogentin, and gentiopicroside (Table 1). Water extracts were also analyzed by MS and this confirms the presence of the above-mentioned compounds characteristic of *G. lutea. *There was no evidence of swertisin and demethylbellidifolin presence in the methanol extract of *G. lutea*. Gentiopicroside is further quantified using UPLC-TUV chromatography (retention time 3.31 min) which revealed that its portion in *G. lutea *methanol extract was 7.79%. This result is in accordance with previously published data [19, 20]. However, the other important bitter component, amarogentin, was detected only in traces using MALDI-TOF, since it is present in lower amount (0.025–0.4%) in the cortex of the root [19, 20].

### 3.2. Inhibition of ALR2 by *G. lutea* Extracts

**Figure 2:**
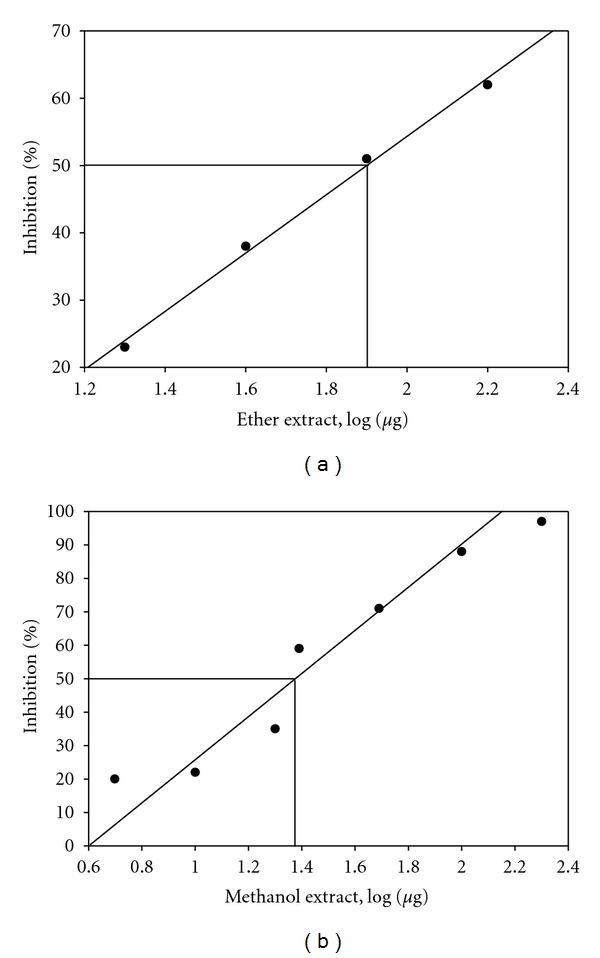
Representative inhibition plots for *Gentiana lutea *ether extract against rat (a) and methanol extract against recombinant human ALR2 (b). ALR2 activity in the absence of the extract was considered as 100%. Data are average of three independent experiments.

All the four extracts of *G. lutea *were tested for their ALR2 inhibitory potential. The representative inhibition curves of rat and human recombinant ALR2 are presented in Figure 2, and the corresponding IC_50_ values of all the extracts are also presented in Table 2. Out of these, while aqueous and ethanol extracts of *G. lutea *moderately inhibited, the methanol and ether extracts significantly inhibited rat lens ALR2 and the recombinant ALR2 (Table 2). The methanol and ether extracts inhibited rat lens ALR2 with an IC_50_ value of 112 *μ*g and 79 *μ*g (Figure 2(a)), respectively. In case of human recombinant ALR2, methanol and ether extract showed an IC_50_ value of 23 *μ*g  (Figure 2(b)) and 36 *μ*g, respectively.

### 3.3. Docking Studies

**Figure 3:**
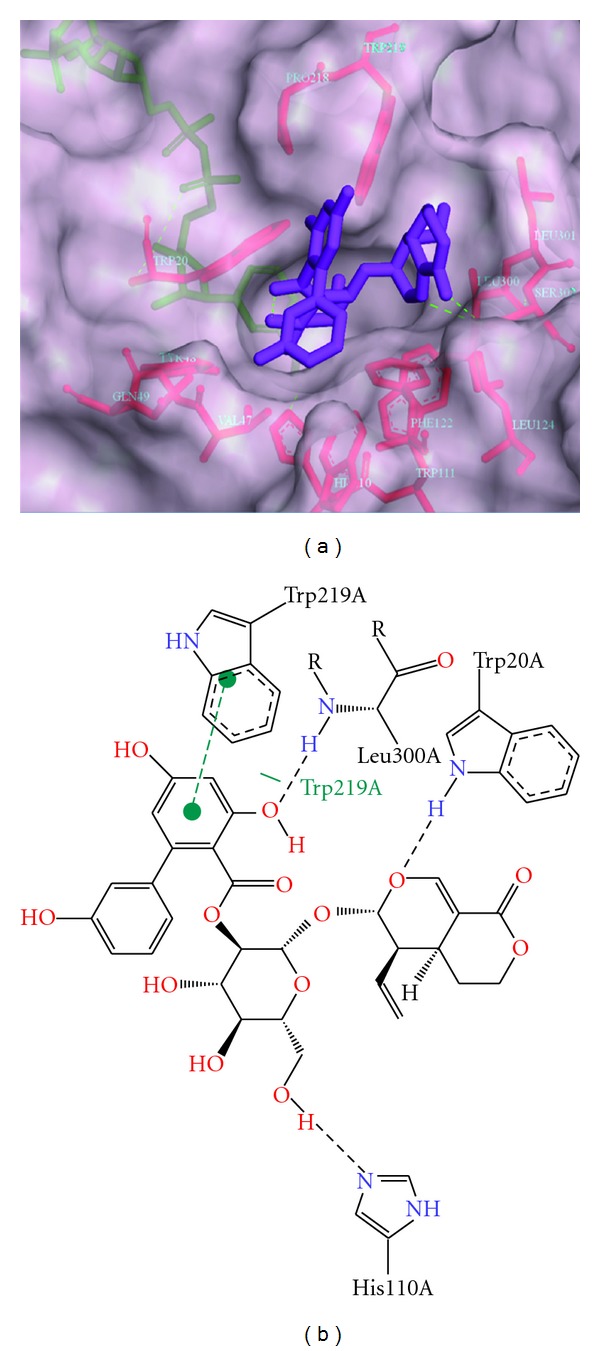
Stereoview of ALR2 docked with amarogentin. (a) Amarogentin docked into the active site of ALR2 and extended towards hydrophobic pocket.**  **(b) Amarogentin docked into active site of ALR2 and depicts its hydrogen bond interaction with residues Trp-20, His-110, and Leu-300 (dotted line) and hydrophobic interactions with Trp-219 (green dotted line).

**Figure 4:**
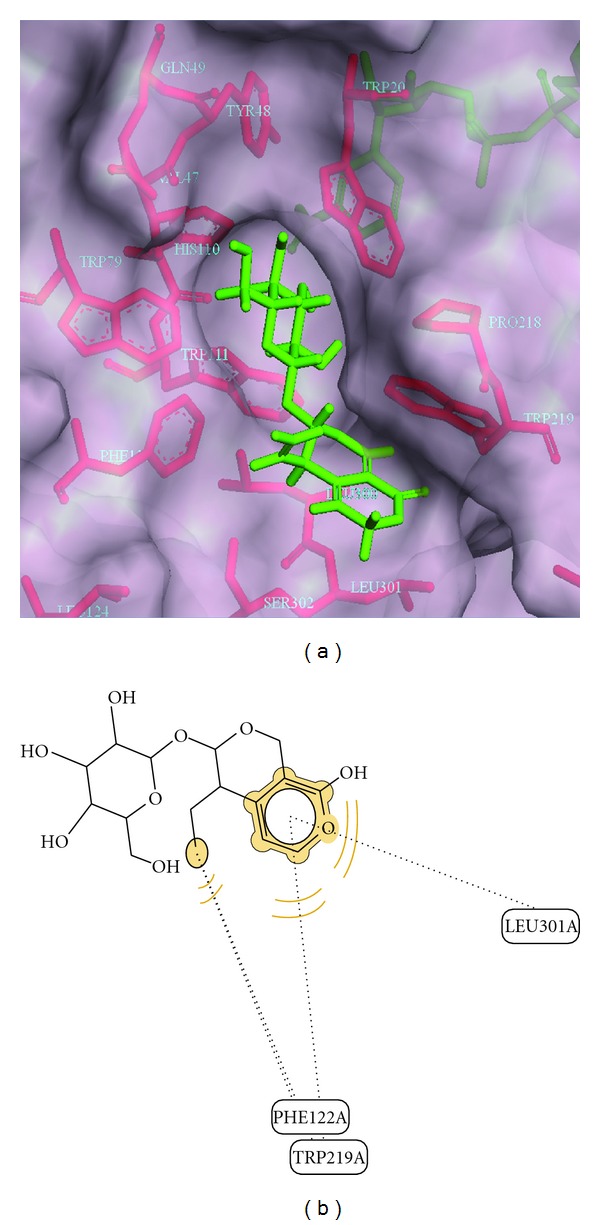
Stereoview of ALR2 docked with gentiopicroside. (a) Gentiopicroside docked into the active site of ALR2 and extended towards hydrophobic pocket.**  **(b) Gentiopicroside docked into active site of ALR2 and depicts its hydrophobic interactions with Trp-219, Phe-122, and Leu-301 (dotted line).

Further, molecular docking studies were carried out with commonly known constituents of *G. lutea *(Table 3). For this, thirteen different compounds selected from various reported studies were used [21–25] which include some compounds identified from our own analysis. Among the thirteen compounds, it was observed that amarogentin showed the highest dock score and possibly interacts with active site residues His-110, Trp-111, Leu-300, and Leu-301. It forms hydrogen bond with Trp-20, His-110, and Leu-300 and hydrophobic interactions with Trp-219. Based on binding of the ligand, the hydrophobic specificity pocket exists in open or closed state. It appears that amarogentin might bind to ALR2 in an open type of conformation because of formation of hydrogen bond with Leu-300 (Figure 3). Compared to the well-known synthetic inhibitor fidarestat which occupied active site of ALR2 with limited contacts, amarogentin extended into the hydrophobic cleft called specificity pocket suggesting effective inhibition of ALR2. Gentiopicroside, which is one of the most abundant compounds found in *G. lutea* roots also showed similar hydrophobic interactions with Trp-219 but did not form any hydrogen bond. Therefore, gentiopicroside binds to ALR2 with the specificity pocket in the closed state (Figure 4).

### 3.4. Suppression of Sorbitol Accumulation

Among human AKRs, ALR2 is unique in its ability to catalyze the NADPH-dependent conversion of glucose to sorbitol [26]. In addition to lens, retina, nerve, and kidney, activation of ALR2 in RBC leads to the accumulation of sorbitol [27]. We have also found a direct correlation between erythrocyte ALR2 and sorbitol levels [28]. Therefore, we assessed accumulation of sorbitol in RBC under high glucose conditions (*ex vivo*) to understand the significance of *in vitro* inhibition of ALR2 by *G. lutea*, particularly its effect on osmotic stress. Incubation of RBC with 55 mM glucose resulted in the accumulation of sorbitol about six-fold higher than the control, whereas presence of all the extracts of *G. lutea* under high glucose conditions leads to reduction in the accumulation of intracellular sorbitol in a dose-dependent manner (Table 4). While the water extract, *G. lutea* showed 35% inhibition at a concentration, of 100 *μ*g, the methanol extract showed similar inhibition at lower concentration that is, 50 *μ*g. Ether extract at the same concentration (50 *μ*g) was more effective than the methanol extract in decreasing sorbitol accumulation by 52%. At 100 *μ*g concentration the ether extract reduced the intracellular sorbitol accumulation almost by 63%. These results not only substantiate the inhibition of ALR2 by *G. lutea* but also indicate their significance in terms of preventing the accumulation of intracellular sorbitol.

## 4. Discussion

Long-term secondary complications are main cause of morbidity and mortality in diabetic patients [2]. Several biochemical mechanisms are involved in the development of these secondary complications. Activation of polyol pathway due to increased ALR2 activity is one of the several mechanisms that have been implicated in the development of various secondary complications of diabetes. Due to its proposed involvement in the development of diabetic complications, ALR2 has been a drug target in the clinical management of secondary complications of diabetes [29]. Structurally distinct compounds such as flavonoids, benzopyrans, spirohydantoins, alkaloids, nonsteroidal anti-inflammatory agents, and quinones have all been shown to inhibit the enzyme with various degrees of efficacy and specificity [4, 7]. Sorbinil, statil, tolrestat, alrestatin, epalrestat, and ALO1576 are some of the well-studied inhibitors that have also been clinically tested. However, to date, none of the currently available synthetic ARIs have proved clinically effective and in fact some have had deleterious side effects. Moreover there is an increased interest in recent times to identify many natural (plant/spice) sources for their therapeutic properties, mainly because most of the plant and plant products are largely free from adverse effects and are being used as a source of diet and traditional medicine.

 The medicinal use of *Gentiana* has a very long tradition. The roots and rhizomes of *G. lutea* showed hepatoprotective [30], antioxidant [31], and anti-inflammatory activity [32] and are used as an appetite stimulant [33]. The medicinal value is due to presence of secoiridoid glycosides and xanthones located mostly in the cortex of the roots of plants belonging to the family of Gentianaceae such as *Gentianella* and *Gentiana* [34]. Hence, we have investigated various extracts of *Gentiana* for their potential to inhibit ALR2.

Results of the present study showing the inhibition of ALR2 by the extracts of *G. lutea* merits attention in many respects. As shown in Figure 2(a), extracts of *G. lutea* inhibited rat lens ALR2, with IC_50_ values ranging 79–260 *μ*g/mL. Although rat lens is known to have the highest ALR2 activity compared to other species, the relevance of inhibition of rat lens ALR2 by *G. lutea* extracts may have limited application to human diabetic complications. Therefore, we have also assessed the inhibitory potential of *G. lutea* extracts against purified human recombinant ALR2 expressed in *E. coli.* Interestingly extracts of *G*. *lutea* inhibited human recombinant ALR2 better than that of rat lens ALR2 with IC_50_ values 23–82 *μ*g/mL (Figure 2(b)). The results with human ALR2 indicate the potential applicability of Gentiana extracts with human target. Secondly, the primary structure of ALR2 displays high similarities with closely related members of AKR superfamily such as human small intestine reductase (HSIR) or AKR1B10. Therefore we next studied the specificity of methanol extract with AKR1B10 and showed its marked specificity towards ALR2 over AKR1B10. Though the ether extract inhibited AKR1B10, the IC_50_ value was ten times higher than ALR2 (400 ± 10 *μ*g), indicating its relative selectivity for ALR2. Also, suppression of sorbitol accumulation in human erythrocytes under high glucose conditions by these extracts is suggestive of translating its impact to *in vivo* conditions.

Although the beneficial impact of strict glycemic control on prevention of diabetic complications has been well established, most individuals with diabetes rarely achieve consistent euglycemia. However, the effects of *Gentiana* on glycemic control (hypoglycemic and antidiabetic) have not been reported and studies are underway to investigate its hypoglycemic potential. Nevertheless, agents that can substantially delay or prevent the onset and development of diabetic complications, irrespective of glycemic control, would offer many advantages. In principle, ARI can be included in this category. Thus, intensive research continues to identify and test both synthetic as well as natural products for their therapeutic value to prevent the onset and/or delay progression of diabetic complications. The results of the present study are a step forward in this direction.

## 5. Conclusions

In conclusion, for the first time we report that extracts of *G. lutea* roots inhibit human recombinant ALR2 *in vitro* and this inhibition appears to be relatively specific towards ALR2 over HSIR. Although, the *in vitro* results per se may have limited implication, these findings provide the directions for exploring the prospects of *Gentiana* extracts to prevent or treat diabetic complications. Therefore, further studies are needed to corroborate these findings in animal models.

## Figures and Tables

**Table 1:** Constituents detected in *G. lutea* methanol extract using MALDI-TOF.

Analyte	Monitored m/z [M + H]^+^
Gentisin	259.5534
Bellidifolin-8-O-glucoside	437.6503
Demethylbellidifolin-8-O-glucoside	423.9371
Isovitexin	433.8520
Swertiamarin	375.6206
Amarogentin	587.1917
Gentiopicroside^∗^	379.7141

^∗^Gentiopicroside pseudomolecular ion is adduct with Na^+^.

**Table 2:** IC_50_ values for ALR2 inhibition by *G. lutea* extracts.

Name of the extract	Rat ALR2 (in *μ*g)	Recombinant ALR2 (in *μ*g)
Water extract	260 ± 20	70 ± 15
Ethanol extract	114 ± 12	82 ± 20
Methanol extract	112 ± 15	23 ± 5
Ether extract	79 ± 12	36 ± 5

Values are mean ± standard deviation of three independent experiments.

**Table 3:** *Gentiana* compounds with their molecular weight and dock score from Accelrys Discovery and GOLD.

S. No	Name of the compound	Mol. wt	Dock score (Discovery)	GOLD score
(1)	Amarogentin	586.54	59.541	29.84
(2)	Gentiopicroside	356.324	57.145	34.20
(3)	Swertianolin	436.366	58.277	33.77
(4)	Loganic acid	376.355	56.399	13.56
(5)	Swertiamarin	374.339	48.984	14.55
(6)	Sweroside	358.34	48.773	27.20
(7)	Isogentisin	258.22	45.786	34.47
(8)	Gentisin	258.22	45.545	31.22
(9)	Bellidifolin	274.225	43.528	29.04
(10)	Bellidin	260.199	40.144	—
(11)	Swertisin	446.404	38.835	—
(12)	Gentianine	175.18	37.292	—
(13)	Gentianadine	149.146	32.51	—

**Table 4:** Effect of *G. lutea* water, methanol and ether extracts on intracellular red cell sorbitol levels.

Group	Water extract	Methanol extract	Ether extract
Control	2.25 ± 0.016	2.47 ± 0.22	2.46 ± 0.25
Glucose 55 mM	12.77* ± 1.21	11.66* ± 1.44	12.16* ± 1.08
Glucose 55 mM + 10 *μ*g extract	8.85^#^ ± 0.25	8.52^#^ ± 1.04	7.25^#^ ± 0.65
Glucose 55 mM + 50 *μ*g extract	—	7.43^#^ ± 0.314	5.74^#^ ± 0.69
Glucose 55 mM + 100 *μ*g extract	8.15^#^ ± 0.32	—	4.49^#^ ± 0.32

Sorbitol levels are expressed as *μ*g/mL RBC. Sorbitol levels were measured in RBC incubated in the presence of normal (5.5 mM) and high (55 mM) glucose for 3 h. *indicates a statistically significant difference from the control group and ^#^indicates a statistically significant difference from the glucose 55 mM group (*P* < 0.05). Values are mean ± standard deviation of three independent experiments.
